# Perceived controllability of a SARS-CoV-2 infection: an investigation of intersectional differences

**DOI:** 10.1186/s12889-022-14848-5

**Published:** 2022-12-17

**Authors:** Till Neugebauer, Diana Wahidie, Fabian Erdsiek, Yüce Yilmaz-Aslan, Patrick Brzoska

**Affiliations:** 1 Health Services Research, Faculty of Health, School of Medicine, Witten/Herdecke University, Alfred-Herrhausen-Straße 50, 58448 Witten, Germany; 2 Dept. of Epidemiology & International Public Health, School of Public Health, Bielefeld University, 33615 Bielefeld, Germany; 3 Dept. of Health Services Research and Nursing Science, School of Public Health, Bielefeld University, 33615 Bielefeld, Germany

**Keywords:** Intersectionality, COVID-19, Perceived controllability, SARS-CoV-2, Health locus of control, Secondary data analysis

## Abstract

**Background:**

The perceived ability to influence an infection with SARS-CoV-2 has an impact on compliance with protective measures. Factors influencing perceived controllability are not yet fully known. The aim of this study was to identify intersectional differences in perceived controllability. Insights into these intersectional differences could help to develop user-centered strategies to improve the acceptance of protective measures.

**Methods:**

Data from the seventh wave of the German Ageing Survey (DEAS) was used to investigate differences in the population regarding the perceived controllability. The role of socio-demographic and socio-economic predictors was investigated using multivariable linear regression modeling. Intersectional differences were examined using interaction terms.

**Results:**

Information on 4,823 respondents aged 46 to 100 years were available, of which 50.9% were female. Migration status (yes vs. no: β = -0.27; 95%-CI = -0.48,-0.06), education level (high vs. low: β = 0.31; 95%-CI: 0.08, 0.55) and employment status (retired vs. employed: β = 0.33; 95%-CI: 0.19, 0.48) were found to be significantly influencing perceived controllability. Interaction effects were found with respect to sex and migration status, with migrant women rating their perceived controllability lower than non-migrant women (β = -0.51; 95%-CI = -0.80, -0.21), while no differences were evident between migrant and non-migrant men (β = -0.02; 95%-CI = -0.32, 0.28). Further intersectional differences were not observed.

**Conclusions:**

The results show that intersectional differences in perceived controllability occur especially between migrant and non-migrant women. Possible causes may lie in language barriers, which in connection with lower health literacy may affect perceived controllability. Dedicated efforts to improve controllability among older adults, those with lower educational attainment and migrant women are warranted.

## Introduction

The COVID-19 pandemic continues to pose far-reaching challenges to countries worldwide. More than 625 million people across the world have contracted COVID-19, and approximately 6.5 million have died from that condition as of Oct 31, 2022 [1]. Although in many countries the proportions of fully vaccinated people are increasing, further waves of infection are to be expected. Therefore, infection control and prevention measures to protect vulnerable populations and prevent or reduce outbreaks will remain important pandemic management strategies for the forseable future [2]. Two of the most effective measures in this regard are the use of face masks and social distancing [3], which have also been a central component of most laws and regulations enacted to mitigate the spread of COVID-19, such as the German Coronavirus Protection Ordinance [4].

To be effective, these measures require acceptance and compliance of the population. Despite initially high levels of approval, a general lack of understanding of the needs and benefits of these measures is increasingly being observed. This leads to reduced acceptance, which is reflected in dismissive or rejective behavior [5]. Results from the Socio-Economic Panel Study (SOEP-CoV) on the impact and consequences of COVID-19 in Germany show that although more than 70 percent of respondents complied with preventive measures, compliance has declined since the beginning of the pandemic [6]. Studies have shown that compliance with recommended measures is associated with health literacy, social cohesion, expected consequences, the willingness to follow rules, and perceived risk [7, 8]. Whether and to what extent recommendations and guidelines are adhered to therefore also depends on the perceived controllability of getting infected with SARS-CoV-2, which is expressed in the individual health-related control beliefs (health locus of control) [9].

First defined by Rotter [10] and further developed by Wallston et al. [11] to address health-related actions, the concept of health locus of control describes how internal and external factors affect individual possibilites of action to achieve a particular goal. Whereas internal control beliefs refer to the extent to which individuals are convinced that they can control health-related events and threats by means of their own behaviour, external control beliefs describe the perceived influence of powerful others, chance, fate or luck over which they have no control [11, 12]. Perceived controllability of infection can be related to both external and internal control beliefs. In research, individuals with a strong external health locus of control have been found to have an increased risk of infection [13, 14]. According to data from the German Ageing Survey, the perceived controllability of the individual risk of infection with SARS-CoV-2 was generally low [15]. Perceived controllability has been reported to be associated with age, health beliefs, self-efficacy and perceived severity as well as education [9, 16, 17]. For example, data from Germany shows that the proportion of those assuming a high level of influence is higher among individuals with a higher level of education than among those with a low level of education (24.3% vs. 21.2%) [15]. However, little is known about potential interactions between influencing factors. It is also unclear to what extent these differences may be influenced by other factors, e.g., SARS-CoV-2 infections among close social contacts. In addition, differences in coping with the COVID-19 pandemic become evident between different population groups. These differences may affect the success of pandemic-related measures and thus have an impact on the health of the population. The aim of the present study was to examine intersectional differences in the perceived controllability of a SARS-CoV-2 infection in Germany. Insights into these associations could help to develop user-centered strategies to improve the acceptance of protective measures.

## Methods

### Data

Data from the seventh wave of the German Ageing Survey (DEAS Short Survey 2020) conducted by the German Centre of Gerontology (DZA) in 2020 were used for the study [18]. The DEAS is a representative cross-sectional and longitudinal survey of people in their second half of life and has been conducted regularly since 1996. It is funded by the Federal Ministry for Family Affairs, Senior Citizens, Women and Youth. Due to the COVID-19 pandemic, the 2020 survey was carried out exclusively as a brief postal survey. During the period from June 8 to July 22, 2020, 4,823 individuals between the ages of 46 and 90 years took part in the survey [18]. Its aim was to obtain an up-to-date overview of life situations and changes experienced as a result of the COVID 19 pandemic in various areas of life. The sample consists of individuals who have already participated in previous DEAS surveys on several occasions. The baseline sample was selected from the resident population in private households, differentiated by age group, sex and region (Eastern/Western part of Germany); stratified random samples were drawn from the population registers.

### Study variables

In the present study, various factors were included to examine intersectional differences in the perceived controllability of an infection with SARS-CoV-2. The outcome of interest was the perceived ability to influence an infection with SARS-CoV-2, measured by means of one Likert item ("To what extent do you feel that you yourself can influence whether or not you contract the coronavirus?") with a 7-point response scale (1 "not at all" to 7 "fully").

The independent variables considered were sex (male; female), migration status (migrants, non-migrants), level of education (ISCED: low; middle; high), age, possible SARS-CoV-2 infection of individuals from the own environment (yes, no, don’t know), partnership status (partner, no partner), occupational status (employed, retired, unemployed), self-rated health status (1 “very good” to 5 “very poor”) and federal state of residence. Individuals who immigrated to Germany themselves or whose parents immigrated to Germany from another country after 1950 were considered to be migrants [19]. Educational level was defined based on ISCED 2011 (International Standard Classification of Education): “low” (ISCED 0–2: without completed vocational qualification and up to a maximum of a graduation degree, which qualifies for a professional qualification), “medium” (ISCED 3–4: with vocational qualifications or qualifications for university or university of applied science entrance) and “high” (ISCED 5–6: with completed university or university of applied science studies). Further definitions can be found in the user manual of the DEAS survey [19].

### Analysis

Descriptive statistics were calculated to describe the sample. Multivariable linear regression was performed to examine the influence of socio-demographic and -economic variables on the perceived ability to influence a SARS-CoV-2 infection. Interaction effects between significant independent variables were included one by one into the model to examine intersectional differences. All analyses were conducted using Stata 16.

## Results

For our study, information on 4,823 respondents aged 46 to 100 years was available (median age = 70), 50.9% of which were female. Respondents from all 16 federal states (“Bundesländer”) of Germany were represented, with the largest proportions being residents of North Rhine-Westphalia (16.4%), Baden-Württemberg (13.5%) and Bavaria (12.4%). 4.5% of the respondents were migrants. 48.3% had a high level of education as defined by the ISCED scale. 26% of the respondents stated to be employees, 70.4% stated that they were retired. 0.8% rated their health as 'very bad', whereas 7.4% reported their health status to be 'very good'. In terms of perceived controllability of infection risk, 5.9% of the respondents had the opinion that they could not influence an SARS-CoV-2-infection 'at all', whereas 6.5% felt they were 'completely' in control (Table 1). The mean value of perceived controllability of getting infected was 4.4.

**Table 1:** Description of the sample (DEAS Short Survey 2020, *n* = 4823)

*Sample characteristics*	*n (%)*
**Sex**	
Male	2366 (49.1%)
Female	2457 (50.9%)
**Migrant status**	
Non-migrants	4598 (95.5%)
Migrants	218 (4.5%)
**Individuals in own environment previously infected with SARS-CoV-2**	
Yes	336 (7.0%)
No	4282 (89.4%)
Don't know	170 (3,6%)
**Educational level**	
Low (ISCED 0–2)	214 (4.4%)
Medium (ISCED 3–4)	2279 (47.3%)
High (ISCED 5–6)	2329 (48.3%)
**Occupational status**	
Employed	1232 (26.0%)
Retired	3332 (70.4%)
Unemployed	166 (3.5%)
**Influence the infection with the Corona virus itself**	
1 Not at all	275 (5.9%)
2	257 (5.5%)
3	591 (12.7%)
4	975 (20.9%)
5	1468 (31.5%)
6	792 (17.0%)
7 Completely	302 (6.5%)
**Self-rated health status**	
1 Very good	355 (7.4%)
2	2305 (48.5%)
3	1703 (35.8%)
4	357 (7.5%)
5 Very poor	36 (0.8%)
**State of residence**	
Schleswig–Holstein	142 (2.9%)
Hamburg	84 (1.7%)
Lower Saxony	399 (8.3%)
Bremen	43 (0.9%)
North Rhine-Westphalia	792 (16.4%)
Hesse	311 (6.4%)
Rhineland-Palatinate	209 (4.3%)
Baden-Württemberg	649 (13.5%)
Bavaria	598 (12.4%)
Saarland	36 (0.7%)
Berlin	168 (3.5%)
Brandenburg	223 (4.6%)
Mecklenburg-Western Pomerania	215 (4.5%)
Saxony	466 (9.7%)
Saxony-Anhalt	319 (6.6%)
Thuringia	169 (3.5%)

The multivariable analysis revealed different factors significantly associated with perceived controllability (Table 2). Migrants (β = -0.27, 95%-CI = -0.48;-0.06) rated the controllability lower than non-migrants; pensioners rated the controllability higher than employed indivduals (β = 0.33, 95%-CI = 0.19; 0.48). Similarly, higher perceived controllability was associated with younger age (β = -0.01, 95%-CI: -0.02; < 0.00), higher education level (β = 0.31, 95%-CI: 0.08;0.55) and better self-rated health status (β = -0.09, 95%-CI = -0.15; -0.03). An examination of interaction effects revealed that migrant women rated the perceived controllability of a SARS-CoV-2 infection lower than non-migrant women (β = -0.51; 95%-CI = -0.80;-0.21), whereas no differences between migrant and non-migrant men could be observed (β = -0.02; 95%-CI = -0.32; 0.28). Further intersectional differences did not become evident.

**Table 2:** Results of multivariable linear regression with perceived controllability of a SARS-CoV-2 infection as the dependent variable. β-coefficients*,* *p*-values and 95% confidence intervals (DEAS Short Survey 2020, *n* = 4823, *n* = 4363 remaining in the final model)

*Independent variable*	*β*	*p-value*	*95%-CI*
**Sex (Ref.: Male)**			
Female	-0.02	0.655	-0.11; 0.07
**Migration status (Ref.: Non-migrants)**			
Migrants	-0.27	**0.013**	-0.48;-0.06
**Age (years)**	-0.01	**0.001**	-0.02; < 0.00
**Individuals in own environment previously infected with SARS-CoV-2 (Ref.:Yes)**			
No	0.13	0.134	-0.04;0.31
Don't know	-0.11	0.473	-0.40;0.18
**Partnership status (Ref.: Partner)**			
No partner	-0.05	0.325	-0.16;0.05
**Educational level (Ref.: Low)**			
Medium	0.06	0.641	-0.18;0.29
High	0.31	**0.009**	0.08;0.55
**Occupational status (Ref.: Employed)**			
Retired	0.33	**< 0.001**	0.19;0.48
Unemployed	0.22	0.079	-0.03;0.47
**Self-rated health status (1 “very good” to 5 “very poor”)**	-0.09	**0.003**	-0.15;-0.03
**Federal state (Ref.: Schleswig–Holstein)**			
Hamburg	0.39	0.057	-0.01;0.79
Lower Saxony	0.04	0.776	-0.25;0.33
Bremen	0.40	0.136	-0.13;0.92
North Rhine-Westphalia	0.12	0.400	-0.15;0.39
Hesse	0.16	0.300	-0.14;0.46
Rhineland-Palatinate	0.08	0.620	-0.24;0.41
Baden-Württemberg	0.11	0.438	-0.17;0.38
Bavaria	0.05	0.707	-0.22;0.33
Saarland	0.42	0.143	-0.14;0.99
Berlin	0.11	0.507	-0.22;0.45
Brandenburg	-0.11	0.495	-0.43;0.21
Mecklenburg-Western Pomerania	-0.02	0.896	-0.35;0.30
Saxony	-0.24	0.093	-0.53;0.04
Saxony-Anhalt	-0.08	0.622	-0.38;0.23
Thuringia	-0.21	0.234	-0.55;0.13

## Discussion

Based on representative survey data for the German population aged 46 years and older, our study shows that lower age, retirement, a high level of education, and better subjective health were associated with higher perceived controllability of getting infected with SARS-CoV-2. In contrast, having a migration status was associated with lower perceived controllability. Differences in perceived controllability of a SARS-CoV-2 infection were particularly evident between migrant and non-migrant women, while no differences were found between migrant and non-migrant men. Other studies report similar findings on low perceived controllability among migrants whereas results on existing sex differences have been inconsistent [17, 20, 21]. These inconsistencies may in part be explained by the heterogeneity of the migrant population as has already been pointed out by previous studies [22]. Possible explanations for our results on these interactions could be limited language skills and the incomplete integration of migrant women into social processes which are considered necessary for the formation of health literacy [23] and constitute an important positive influence on perceived control over one’s own health [24]. The lack of culturally and linguistically sensitive information can therefore contribute to a lower perceived controllability of infections.

The results of the analysis show that education also played an important role in influencing perceived controllability in this context. This finding is in line with previous research [25, 26]. It can be explained by the association between high education levels and high health literacy [27], which could also suggest that people with a high level of perceived control are more concerned with accumulating information about the risk of contracting COVID-19 and its consequences. The subjective importance of adhering to protective measures therefore seems to be rated higher among individuals with a higher level of education than among those with a lower level of education [28]. However, this does not be rule out that individuals with lower education levels also recognize the drastic threat posed by the pandemic and take action accordingly. In this context, user-centered strategies to convey relevant information on health behavior during a pandemic are essential in order to reach people with lower and higher education equally as well.

Another result of this study was how the age of the respondents is related to perceived controllability. The younger the study participants were, the better they assessed their controllability. This confirms previous research on control beliefs showing that older individuals are more likely to consider events and health threats be influenced by powerful others and chance [19, 23]. In terms of COVID-19, better perceived controllability in younger individuals could, however, be explained by individuals’ awareness to having a lower risk of a severe course of COVID-19 compared to people of older age [29]. Nevertheless, younger individuals may still perceive a strong threat from COVID-19. Different studies show that younger individuals feel equally or even more threatened in their own emotional well-being, finances and work goals by COVID-19 than older individuals [15, 30]. This can be attributed to potential consequences of the pandemic, such as job loss or social disengagement [31]. The fear of these consequences may contribute to increased precautions taken by younger adults, who are primarily trying to protect others [15, 32]. This in turn may result in a higher perceived controllability of infection with SARS-CoV-2 among younger adults. Interestingly, our results show contrasting associations between perceived controllability and occupational status. Retired individuals in our study perceived higher controllability than employed individuals. A possible explanation could be that social life and connections change with retirement. The reduction in daily, work-related contacts may give retirees a sense of security that leads to increased controllability. However, this may also results in less information seeking behavior related to COVID-19, which could decrease if regular interactions with co-workers or others are reduced. In addition, the ability to use new ways to communicate, like digital devices, is less common among older individuals [33]. Health-related information should therefore be distributed via different channels to ensure better accessibility.

We also found a significant association between the subjective health status and perceived controllability. In this case, a low self-rated health was associated with a lower perceived controllability. This could be due to additional burdens caused by existing diseases or physical and psychological activities to control one’s own state of health. Perceived controllability may also be assessed differently if individuals have contracted COVID-19 themselves [12]. Since only 0.4% of respondents in our study reported a previous or recent COVID-19 infection, we could not examine that aspect further.

High perceived controllability over infection risk suggests an internal health locus of control, which also by previous research has been found to be associated with higher resilience, lower overall healthcare utilization and better self-rated health [34]. Internal health locus of control has also been found to promote medication adherence and adherence to exercise programs in patients. This suggests that higher perceived control does not generally predict lower adherence to protective measures [35, 36]. Further research is needed on how these suggestions can be applied on infectious diseases like SARS-CoV-2.

### Strengths and limitations

One major strength of our study is the use of a large and representative dataset, which makes it possible to investigate how adults in Germany perceive the controllability of the COVID-19 pandemic and their subjective influence on being infected as well as which factors influence this perception. However, this does not necessarily predict adherence to protective measures. The survey data we used only included adults aged 46 years and older, limiting the generalizability of our findings. In addition, when interpreting the results, the period during which the survey was conducted must be taken into account, as hardly any restrictive infection control measures were in place during this time. Daily incidence rates were also low, which may have led respondents to rate controllability high because they did not perceive an immediate threat.

Most population surveys are prone to some degree of sampling bias [37]. For the German Ageing Survey, investigations have shown that this bias can be regarded small, as the survey’s distribution of demographic and socioeconomic characteristics corresponds to that of administrative data on the population in Germany [38]. However, with regard to the investigation of intersectional differences, it must be noted that migrants were underrepresented in the sample. This is presumably due to the fact that the survey was only conducted in German language, thus excluding migrants with little knowledge of the German language. Given that limited language skills are often associated with lower control beliefs, it can be assumed that the disparities between migrants and non-migrants were underestimated in the present study.

A further limitation of this study is a possible bias resulting from residual confounding. In order to allow coverage of as many areas as possible given the short format of the survey, the respective topics were limited in scope. Because of this limitation, only few independent variables could be considered in our analysis. Further investigations focusing on subjective health, the handling of health information as well as on possible other factors influencing perceived controllability are therefore warranted.

## Conclusions

The results obtained in this study provide an overview of the association between the perceived controllability of a SARS-CoV-2 infection and the role of various socio-demographic and health-related factors. Although the investigation has been conducted in a particular period of the pandemic, it highlights the need to address low levels of perceived controllability among migrants, among lower education groups and among older individuals. With respect to migration, it illustrates the need for an intersectional perspective by highlighting that differences in the perceived controllability exist for migrant and non-migrant women while they do not exist for men. Simultaneously, some of the factors associated with perceived controllability are also associated with a higher vulnerability towards specific diseases and general health impairment as previous research shows. Therefore, additional efforts which take into account the heterogeneity of the population groups as well as the role of intersectional differences are necessary. Consequently, comprehensive information campaigns on the consequences of a possible infection with SARS-CoV-2 and the effectiveness of different preventive measures are warranted.

## Data Availability

The data presented in this study can be obtained free of charge from the Research Data Centre of the German Ageing Survey at [doi:10.5156/DEAS.2020.M.001].
